# Treatment of Aqueous Amoxicillin Solutions with Sunlight Using a Pelletized Macrocomposite Photocatalyst

**DOI:** 10.3390/ma18071394

**Published:** 2025-03-21

**Authors:** Saad Slimani Tlemcani, Zenydia Marín, J. Arturo Santaballa, Moisés Canle

**Affiliations:** React! Group, Department of Chemistry, Faculty of Sciences & CICA, Universidade da Coruña, E-15071 A Coruña, Spain; saad.slimanitlemcani@udc.es (S.S.T.); zenydia.marin@udc.es (Z.M.); arturo.santaballa@udc.es (J.A.S.)

**Keywords:** amoxicillin, antibiotic, copper doping, cobalt doping, kaolin, TiO_2_-P25, sunlight, photodegradation, reaction mechanism, reuse, macrocomposite

## Abstract

We report on the preparation and characterization of a cost-effective, durable, and reusable macrocomposite, prepared in the form of pellets and designed for the photodegradation of water pollutants, using amoxicillin (AMX) as a model compound. Using the wet impregnation method, kaolin clay and TiO_2_-P25 composites were doped with copper (Cu^2+^) and cobalt (Co^2+^). The produced materials were characterized by SEM-EDS, XRD, XRF, textural property analysis, and their potential lixiviation of components by ICP-MS. The photodegradation efficiency under solar irradiation was evaluated by varying the acidity of the medium, the concentration of AMX, and the amount of catalyst. The performance of the recycled photocatalysts was also studied. The photodegradation of AMX was monitored by UV–Vis and UV–Vis/HPLC spectrophotometry. The optimal formulations, Cu (0.1%)/TiO_2_ and Co (0.1%)/TiO_2_, achieved up to 95% degradation of 5 mg·L^−1^ AMX in 3 h at pH 5.9, with a catalyst loading of 1 g·L^−1^. The Cu-doped material showed a slightly faster reaction rate and higher total-organic-carbon removal (80.4%) compared to the Co-doped material (59%) under identical conditions. The same photodegradation intermediates were identified by LC-MS/MS for both doped macrocomposites, and a reaction mechanism is proposed. These macrocomposites showed efficient and consistent recyclability over more than five reuse cycles, showing their potential to be used for antibiotic pollution abatement in water-treatment facilities.

## 1. Introduction

The preservation of water, an essential resource for life on Earth, is a global priority. However, human activities have significantly deteriorated water quality, with pollution by antibiotic drugs emerging as a critical concern because of the possible emergence of antimicrobial resistances, among others. Hospital, pharmaceutical, and residential wastewater often contain residues of antibiotics that adversely affect aquatic ecosystems and pose potential risks to human health. To mitigate the environmental and health impacts of a growing pollution, advanced techniques have been developed for the oxidation and degradation of antibiotics in water. These methods aim to transform antibiotics into safer molecules or, ideally, eliminate them, thereby reducing their concentration in water sources. Among these strategies, advanced oxidation processes (AOPs) have demonstrated effectiveness in removing a broad range of organic contaminants from water and wastewater through reaction with hydroxyl radicals (HO^•^) [1,2].

Photocatalysis, one of the most promising AOPs, has been widely investigated using various photocatalysts such as Al_2_O_3_, ZnO, Fe_2_O_3_, and TiO_2_ [3]. Among them, TiO_2_ stands out due to its chemical stability, non-toxicity, high photocatalytic activity, and cost effectiveness. TiO_2_ is typically used in the form of an anatase–rutile mixture, commercially known as TiO_2_-P25 (Evonik), which is particularly efficient [4]. TiO_2_-P25, activated by solar irradiation, is a reliable solution for degrading persistent organic pollutants in water through HO^•^ radical generation. Efforts to enhance the photocatalytic performance of TiO_2_-P25 under solar irradiation have included modifications with metal dopants.

We have previously shown that transition metal-impregnated TiO_2_ is effective in photocatalytic applications. Cu-modified TiO_2_ demonstrated improved photocatalytic activity under Vis light, achieving over 95% total organic carbon (TOC) removal for phenol degradation. This improvement is due to a reduced bandgap energy and better charge-carrier separation [5]. Similarly, the Co-modified TiO_2_ performed exceptionally well under near-UV–Vis radiation, achieving 96% TOC removal for phenol degradation. This success can be attributed to the creation of oxygen vacancies and inter-band states that allowed for greater light absorption in the visible spectrum [6]. These results underscore the potential of metal-modified TiO_2_ as highly effective photocatalysts for environmental processing.

**Scheme 1:**
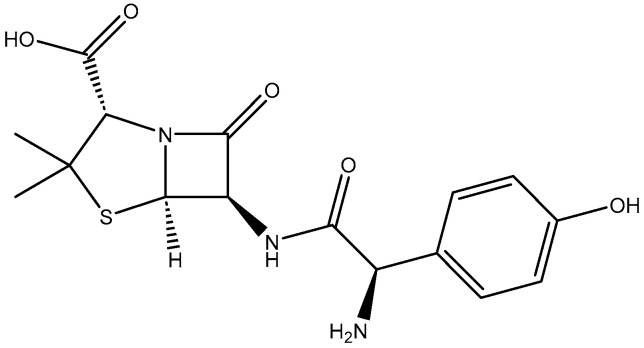
Amoxicillin structure.

Here, we summarize our investigations on the photocatalytic degradation of amoxicillin (AMX), a widely used beta-lactam antibiotic, under sunlight irradiation. AMX, (2S,5R,6R)-6-[[(2R)-2-amino-2-(4-hydroxyphenyl)acetyl]amino]-3,3-dimethyl-7-oxo-4-thia-1-azabicyclo[3.2.0]heptane-2-carboxylic acid, belongs to the penicillin family and contains a beta-lactam ring and a thiazolidine ring (Scheme 1). It is effective against a broad spectrum of Gram-positive and Gram-negative bacteria [7].

To enhance the efficiency of AMX degradation, we prepared a series of macrocomposite photocatalysts based on kaolin and TiO_2_-P25 (90% TiO_2_-P25 and 10% kaolin), impregnated with different weight percentages of copper (Cu^2+^) and cobalt (Co^2+^) (0.1%, 0.5%, 1%). These materials were characterized using X-ray diffraction (XRD), diffuse reflectance spectroscopy (DRS), scanning electron microscopy (SEM), and other techniques. We optimized key parameters influencing amoxicillin photodegradation, such as pH, amoxicillin concentration, and photocatalyst dosage. The degradation process was monitored using high-performance liquid chromatography (HPLC), UV–Vis spectroscopy, and total organic carbon (TOC) analysis, while photodegradation products were identified via HPLC-MS.

Promising results were achieved with both the Cu^2+^ and Co^2+^-modified photocatalytic materials, attaining approximately 86% AMX degradation within 180 min under sunlight irradiation. Verma and Haritash, studying the photocatalyzed degradation of AMX, found a degradation of 75% [8]. These findings highlight the potential of metal-doped TiO_2_-P25 macrocomposites for efficient antibiotic degradation in water-treatment applications [9,10,11].

## 2. Experimental Procedure

### 2.1. Materials

TiO_2_-P25 was acquired from Evonik (ca. 70% anatase, 30% rutile, and a tiny proportion of amorphous phase), with a surface area of 55 ± 15 m^2^·g^−1^ [12] and a particle size of ~30 nm [13]. Copper (II) sulfate pentahydrate (CuSO_4_·5H_2_O) (≥98%, Sigma, St. Louis, MO, USA), Cobalt (II) sulfate heptahydrate (CoSO4·7H2O) (≥97%, Aldrich), and AMX (C_16_H_19_N_3_O_5_S) (99.5% Sigma-Aldrich, St. Louis, MO, USA) were purchased and used without further purification. Acetonitrile (HPLC grade) was purchased from J.T. Baker (Center Valley, PA, USA). Some studies employed O_2_ gas with a purity of at least 99.995%. The distilled water was taken from a Millipore device (Milli-Q water, Burlington, MA, USA), with a resistivity of 18.2 MΩ at 298.0 K and a total organic carbon (TOC) of less than 5 μg/L.

### 2.2. Catalyst Synthesis

The immobilization of metals onto the photocatalyst was achieved using the incipient wetness impregnation method. A kaolin clay and TiO_2_-P25 composite was prepared by dissolving the required amount of metal salt in distilled water; the clay-TiO_2_ mixture was then gradually added to the solution under constant stirring. The resulting suspension was stirred continuously for 24 h at 50 °C to ensure a uniform distribution of the metal ions throughout the composite.

The mixture was then manually extruded through a syringe with a 2 mm inner-diameter tip to form elongated, spaghetti-like rolls, and deposited on a clean aluminum sheet. These rolls were dried in an oven at 90 °C for 24 h. Subsequently, the dried photocatalysts were calcined at 600 °C for 4 h, with a ramp rate of 10 °C·min^−1^. The calcined materials, hereafter called M (X%)/TiO_2_, were finely ground, where M (X%) denotes the specific metal (M = Cu or Co) and its mass percentage (*X* = 0.1%, 0.5%, or 1%).

To remove loose particles, the photocatalysts were rinsed thoroughly with distilled water and then dried again at 90 °C. To evaluate Cu^2+^ and Co^2+^ potential leaching, the samples M (0.1%)/TiO_2_ were immersed in water with stirring for 24 h. The resulting solution was filtered and analyzed using inductively coupled plasma mass spectrometry (ICP-MS) to determine Cu^2+^ and Co^2+^ concentration, 1476 μg/L and 10,950 μg/L at pH 5.9, respectively, indicating a slight leaching process. Copper was found in the leaching water below the limit of 2,0 mg/L established by the EU, which would allow for the use of the Cu (0.1%)/TiO_2_ macrocomposite for water treatment [14]. Though no maximum admissible limit of cobalt has been established by the UE or WHO, the US EPA has established a reference maximum admissible limit at 0.1 mg/L, well below the lixiviated concentration achieved in this experiment, a clear indication that the Co (0.1%)/TiO_2_ macrocomposite is not mechanically stable and further work on its stabilization would be needed prior to scaling up its use [15].

### 2.3. Photocatalyts Characterization

The morphology of TiO_2_-P25, Cu (X%)/TiO_2_, and Co (X%)/TiO_2_ photocatalysts was analyzed using a JEOL JSM-6400FEG scanning electron microscope (JEOL Ltd., Tokyo, Japan) equipped with an energy-dispersive X-ray spectroscopy (EDS) system. Prior to imaging, the samples were coated with a thin layer of gold (~2 nm) and mounted on small pieces of amorphous carbon.

The surface morphology of the photocatalysts was further examined using a Jeol-JEM-1010 microscope (JEOL Ltd., Japan) operated at an accelerating voltage of 80 kV. For TEM analysis, a few drops of a nanoparticle suspension were deposited onto carbon-coated copper grids (200 mesh) and allowed to air-dry before imaging.

X-ray diffraction (XRD) analysis was conducted using a Bruker Siemens D5000 diffractometer (Bruker Corporation, Billerica, MA, USA) equipped with Bragg–Brentano geometry and a θ/2θ configuration. The system incorporates a graphite monochromator and features advanced optics, including 2° primary and secondary Soller slits, a variable divergence slit, a 1 mm receiving slit, a 0.2 mm monochromator slit, and a 0.6 mm detector slit. A scintillation counter served as the detector. Data acquisition was performed over a 2θ range of 2–80° with a step size of 0.050° and a dwell time of 2.5 s per step. The DiffracPlus software (v. 8.0.0.2, Socabim, Billerica, MA, USA) was employed for data processing and analysis.

N_2_ adsorption/desorption BET isotherms were obtained at 77.4 K (Tristar II Plus-Micromeritics, Norcross, GA, USA), between P/P_0_ = 0.1 and 1.0. The relative pressure range used to calculate the BET-specific surface area was P/P_0_ = 0.05–0.3. “MicroActive for TriStar II Plus” v. 2.03 (Micromeritics, Norcross, GA, USA) software was utilized for instrument control, data acquisition, and data processing.

The UV–Vis diffuse reflectance spectra (200–800 nm) of solid photocatalysts were measured using a JASCO V-560 UV-Vis spectrophotometer (Hachioji, Tokyo, Japan) with a double monochromator and double-beam optical system, as well as an integrating sphere attachment (JASCO ISV-469, Hachioji, Tokyo, Japan). Reflectance spectra were transformed into equivalent absorption Kubelka-Munk units.

### 2.4. Photocatalytic Degradation Procedure

Photocatalytic degradation experiments were carried out on sunny days from 10:30 a.m. to 2:30 p.m. at A Coruña, Spain (43°19′36″ N, 8°24′33″ W). AMX solution was initially prepared by adding 5 mg of AMX to 1 L of deionized water. In the photocatalytic degradation experiment, the effect of operational parameters such as initial pH (3.3, 5.9, and 8.6), catalyst dose (0.1 and 1.0 g/L), and initial AMX concentration (1–9 mg/L) were studied. The experiment was conducted at temperatures ranging from 19 to 24 °C, and the solution pH was modified to the desired level, when needed, using 1M HCl(aq) and 1M NaOH(aq).

The photocatalytic degradation experiment was carried out as follows: 1 g of the photocatalyst was dispersed in 50 mL of an aqueous AMX solution with an initial concentration of 5 mg/L. The suspension was then exposed to sunlight to initiate the photocatalytic reaction. Samples were collected at specific time intervals during irradiation to monitor AMX photodegradation. The collected samples were analyzed using HPLC to determine the extent of AMX degradation. The degradation efficiency (% degradation) was calculated as follows:

$$\%\;AMX\;degradation=\frac{{\left[AMX\right]}_{0}-{\left[AMX\right]}_{t}}{{\left[AMX\right]}_{0}}\cdot 100$$

where [AMX]_0_ represents the initial AMX concentration and [AMX]_t_ is the remaining antibiotic concentration at time (t) [16,17]. Fitting the first-order equation to the kinetic data allowed us to obtain the corresponding pseudo-first-order rate constants.

The photocatalytic degradation efficiency was calculated based on the initial AMX concentration. AMX was monitored by measuring the UV–Vis absorbance at 226 nm and 272 nm, using a Biochrom Libra S70 spectrophotometer (Cambridge, UK), and by UV–Vis HPLC analysis also at 226 and 272 nm, in a Thermo Fisher apparatus equipped with a 6000 LP UV detector (Waltham, MA, USA), an AS 3000 automatic sample, and a P4000 solvent pump (Waltham, MA, USA). The Kromaphase C18 column (Waltham, MA, USA) is 4.6 mm × 150 mm × 5 µm was used, with an injected volume of 50 µL, a flow rate of 1.0 mL·min^−1^, at 25 °C, with acetonitrile buffer solution in pH = 4.45 (25:75, *v*/*v*) as mobile phase. The TOC removal efficiency was determined by a Shimadzu TOC-5000A analyzer (Kyoto, Japan).

Photoproducts were detected by HPLC/MS (Thermo Scientific LTQ Orbitrap Discovery tools (Waltham, MA, USA) using an electrospray interface in positive ion mode (ESI+). Kromaphase 100 C18 column (Waltham, MA, USA) (150 mm × 5 μm) was used, operated at 25 °C, with elution solvents A (H_2_O 0.1% H-COOH) and B (ACN 0.1% H-COOH) at flow rate of 0.4 mL/min. The gradient was as follows: 0–2 min, 97–97% A and 3–3% B; 2–5 min, 97–80% A and 3–20% B; 5–12 min, 80–20% A and 20–80% B; 12–14 min, 20–20% A and 80–80% B; 14–15 min, 20–97% A and 80–3% B; 15–20 min, 97–97% A and 3–3% B.

## 3. Results and Discussion

### 3.1. Catalyst Characterization

The efficiency of a photocatalyst depends on the surface and structural properties of the semiconductor, such as its crystalline structure (facets exposed), surface area, particle-size distribution, porosity, band gap, and the density of hydroxyl groups on the surface [18]. The use of doped metals such as copper (Cu) and cobalt (Co) in photocatalysts for the removal of AMX improve the photocatalytic activity of the base material, TiO_2_, by altering the electronic and structural characteristics. Low doping concentrations (e.g., 0.1% to 1%) are generally enough to produce desirable changes in the photocatalyst (narrowed bandgap, improved charge separation, and increased ROS generation) without overloading the system [19,20,21]. Over-doping (>1%) generates an excess of defect sites within the photocatalyst lattice, that can promote e^−^/h^+^ recombination, thus reducing the overall efficiency of the photocatalytic process [22,23,24].

#### 3.1.1. Scanning Electron Microscopy (SEM) and Energy-Dispersive Spectroscopy (EDS)

**Figure 1:**
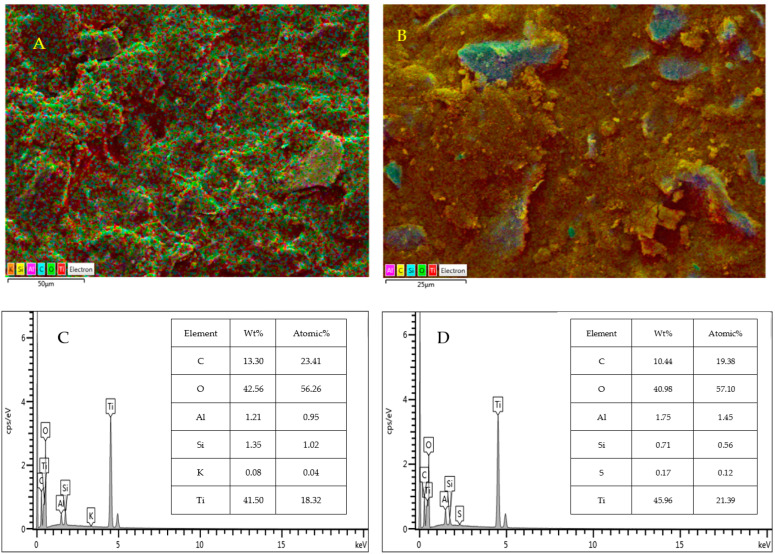
SEM-EDS micrographs of raw Cu (0.1%)/TiO_2_ (**A**,**C**) and Co (0.1%)/TiO_2_ (**B**,**D**) calcined at 600 °C.

The SEM and EDS analyses of M (0.1%)/TiO_2_ are displayed in Figure 1. The micrographs of Cu (0.1%)/TiO_2_, Co (0.1%)/TiO_2_ show the presence of irregularly shaped particles with different sizes (Figure 1A), and of some large crystalline particles (Figure 1B). M (0.1%)/TiO_2_ surfaces remain stable, as they exhibit no cracks and are characterized solely by the presence of cavities. EDS analysis of M (0.1%)/TiO_2_ shows the dominance of Ti, O, and C, and to a lesser extent Al and S were also detected, but in lower amounts (Figure 1C,D).

#### 3.1.2. X-Ray Diffraction (XRD) and Fluorescence (XRF)

**Figure 2:**
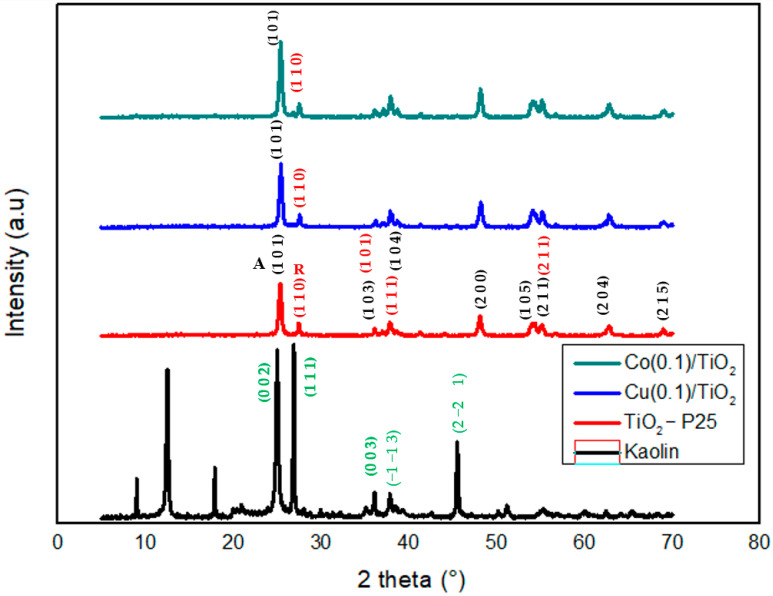
X-ray diffraction patterns of clay (kaolin) (black),TiO_2_-P25 (red), Cu (0.1%)/TiO_2_, (blue), and Co (0.1%)/TiO_2_ (green). (A) anatase; (R) rutile.

The crystalline structure of the as-synthesized M (0.1%)TiO_2_ samples was evaluated using XRD analysis (Figure 2). The seven prominent diffraction peaks (2θ: 25.25°, 37.52°, 48.02°, 53.58°, 54.88°, 62.61°, and 75.07°—red diffractogram) are attributed to the anatase phase of TiO_2_ in pristine TiO_2_-P25, as identified by JCPDS No. 00-021-1272 [25,26]. The anatase phase predominates (80% anatase and 20% rutile) at low processing temperatures, whereas the rutile one becomes dominant at higher temperatures, typically above 600 °C (it is commonly formed through high-temperature calcination of the anatase phase) [27].

Cu (0.1%)/TiO_2_ and Co (0.1%)/TiO_2_ diffractograms (Figure 2) do not show new peaks due to the presence of Cu and Co after impregnation, which may indicate that the low amount of Cu and Co is well disseminated into TiO_2_ lattice. Some researchers [26,28] suggested that no dopant peaks are detected due to the low dopant load. In spite of the absence of new peaks, doping with Cu and Co increases the intensity of the existing ones (Figure 2). Some studies showed similar behavior, but the increased intensity was related to an improvement in crystallinity [29] a phenomenon that may be attributed to a size mismatch between Cu or Co and TiO_2_, with a beneficial effect on the TiO_2_ crystallinity.

Table 1 shows the result of the fluorescence X-ray analysis (XRF) of the as-prepared photocatalytic materials. Cu (0.1%)/TiO_2_ and Co (0.1%)/TiO_2_ are predominantly composed of TiO_2_, SiO_2_, Al_2_O_3_, and Fe_2_O_3_, which account for 97.70% and 98.85%, respectively, of the total weight of the photocatalysts. The small percentage of Fe_2_O_3_ and SiO_2_ observed is attributed to the presence of kaolin -Al_2_Si_2_O_5_(OH)_4_-.

#### 3.1.3. Textural Properties

**Figure 3:**
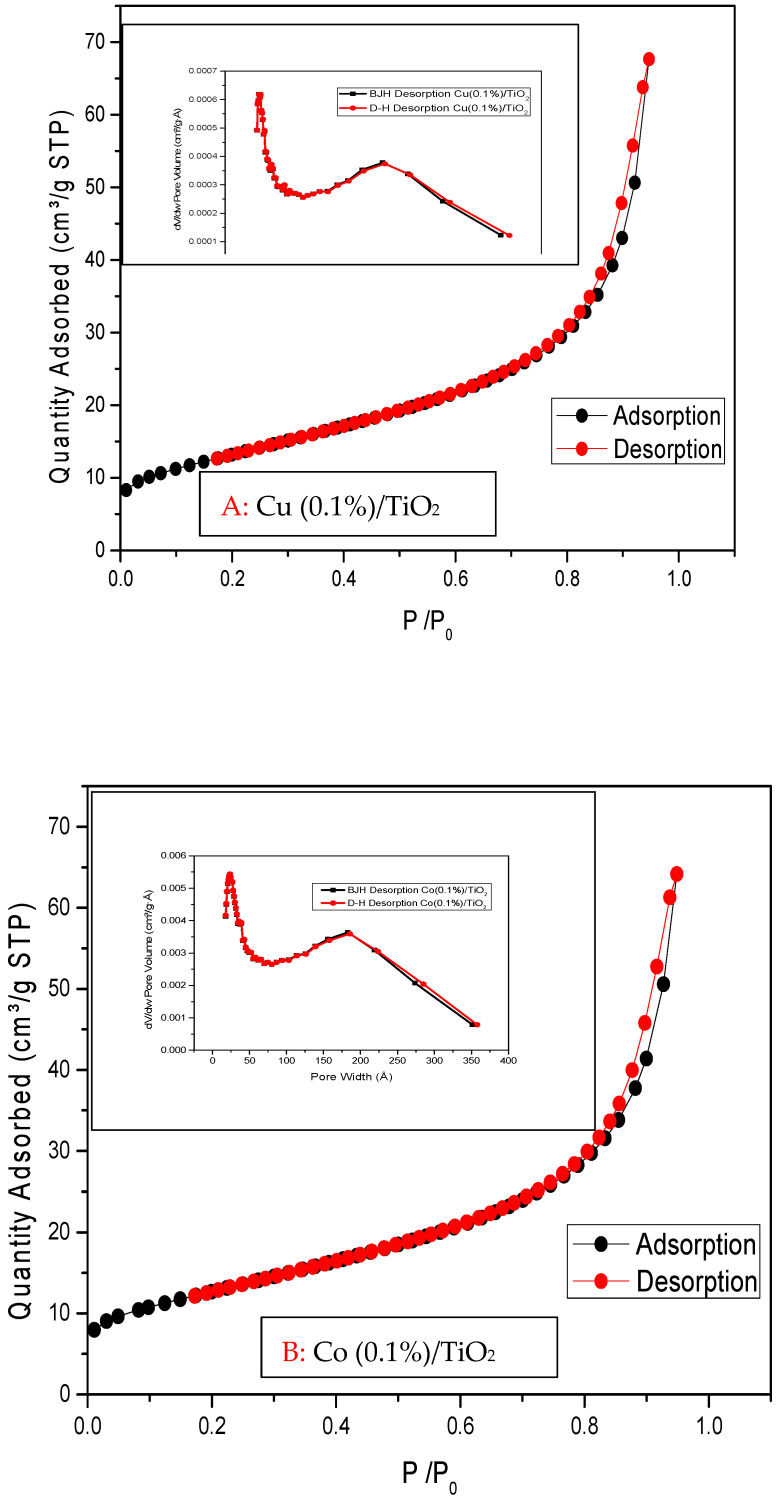
N_2_ adsorption–desorption isotherm of (**A**) Cu (0.1%)/TiO_2_ and (**B**) Co (0.1%)/TiO_2_ photocatalyst. Inset: pore-size distribution.

The N_2_ adsorption–desorption isotherms of Cu (0.1% TiO_2_) and Co (0.1% TiO_2_) and the corresponding pore-size distribution are shown in Figure 3; both isotherms correspond to type IV according to the IUPAC classification, featuring an H3-type hysteresis loop observed near the high-relative-pressure region (0.9 < P/P_0_ < 1), characteristic of a typical mesoporous structure [30].

The porous microsphere M (X%)/TiO_2_ stacking forms cavities with a pore-size distribution ranging from 1 to 5 nm, consistent with the SEM analysis. The isotherm was utilized to calculate the BET surface area (SBET, multipoint) and to evaluate the textural properties based on the Barrett, Joyner, and Halenda (BJH) model [31] (Table 2).

The Cu-doped photocatalyst exhibits a slightly higher BET surface area and a larger BET constant (C), reflecting an increased affinity for N_2_. While the median pore width remains comparable between the two photocatalysts, the Cu-doped sample shows a slightly greater maximum pore volume. On the other hand, the Co-doped photocatalyst has a marginally larger average particle size, despite both materials displaying similar median pore widths. These properties suggest potential effectiveness in pollutant adsorption and UV light absorption.

The observed textural characteristics differ from those observed in Cu and Co samples prepared via hydrothermal process (Table 2), where the type IV isotherm exhibits a significant H3 hysteresis loop, but a lower P/P_0_ range (0.4–0.8) [32,33].

The pore-size distribution (PSD) reveals a maximum at 23 Å for Cu (0.1%)/TiO_2_ and 22 Å for Co (0.1%)/TiO_2_, both at the lower limit of the mesoporous range.

Adsorption of AMX on both types of pelletized composites was studied in the dark. The adsorption–desorption equilibrium was attained at 120 and 180 min, respectively, for Cu (0.1)/TiO_2_ and Co (0.1)/TiO_2_ (Table 3). The adsorption followed first-order kinetics, with rate constants k_ads,Cu (0.1)/TiO2_ = 2.54 × 10^−3^ s^−1^ and k_ads,Co (0.1)/TiO2_ = 1.79 × 10^−3^ s^−1^, respectively. The adsorption experiment also revealed that, under dark conditions, Cu (0.1%)/TiO_2_ and Co (0.1%)/TiO_2_ adsorbed 19% and 13% of the initial AMX concentration, respectively, after 180 min, from which the corresponding adsorption–desorption equilibrium constants can be estimated as K_a-d Cu (0.1)/TiO2_ = 0.23 L·g^−1^ and K_a-d Co (0.1)/TiO2_ = 0.15 L·g^−1^, respectively. These values confirm the system is not far from equilibrium, as expected in the framework of the Langmuir approach.

### 3.2. Photodegradation of AMX upon Sunlight Irradiation

The photocatalytic degradation of AMX in aqueous solution ([AMX]_0_) = 5 mg/L) under solar irradiation was monitored using Cu (X)/TiO_2_ and Co (X)/TiO_2_ macrocomposite pellets with different metal concentrations (X = 0.1%, 0.5%, 1.0%).

**Figure 4:**
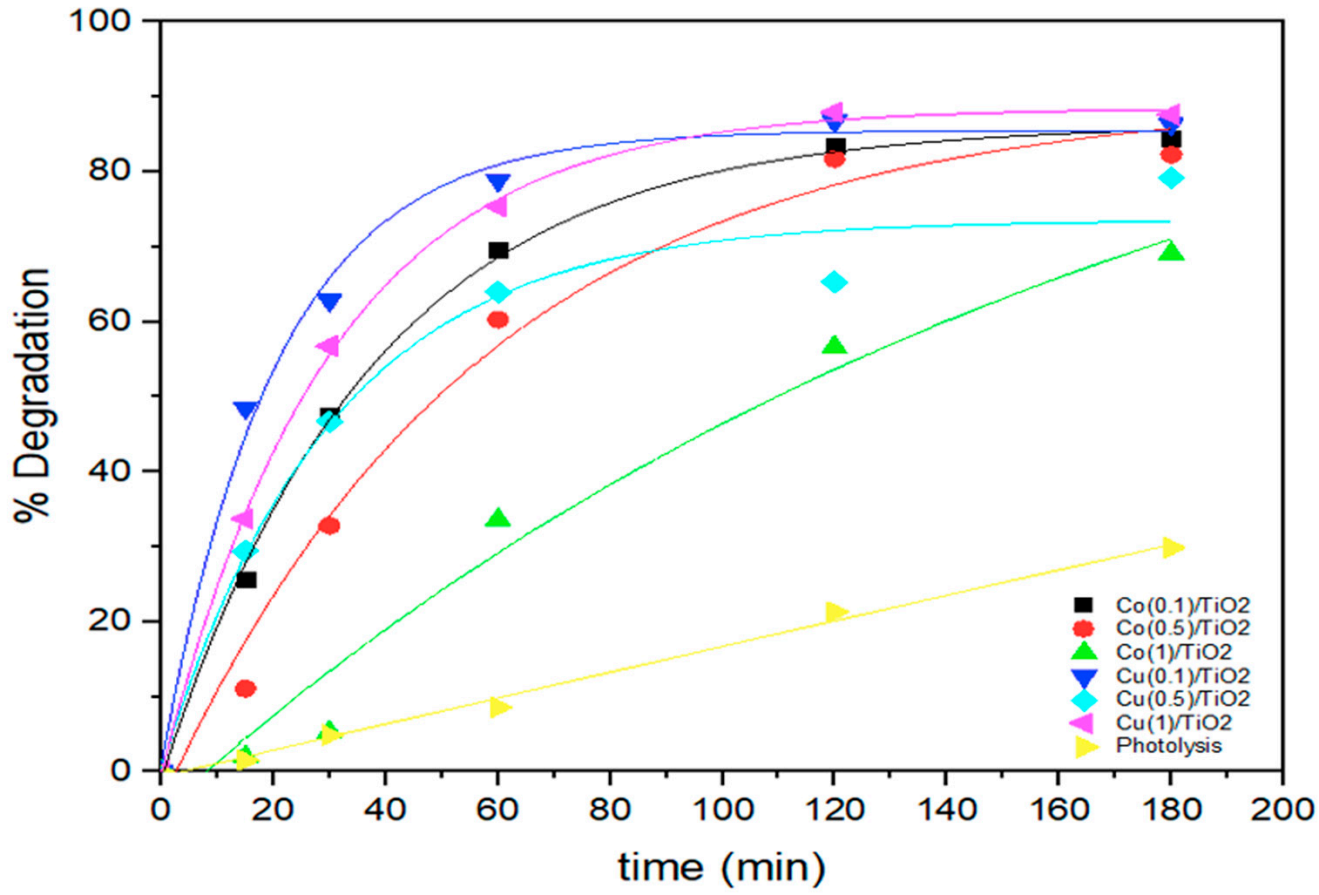
AMX photocatalytic degradation using different Co (X%)/TiO_2_ and Cu (X%)/TiO_2_ macrocomposites (pellets) under sunlight. [AMX]_0_ = 5 mg/L; photocatalyst load = 1 g/L; pH = 5.9; T = 19 °C.

Cu (X) and Co (X) catalysts, with varying metal concentrations, were tested for their efficiency in degrading AMX under sunlight exposure [34]. The most effective concentrations were 0.1% and 0.5% for Co, and 0.1% and 1% for Cu (Figure 4 and Table 4). Both catalysts exhibited similar degradation trends, achieving comparable efficiencies at 120 min and beyond, ultimately reaching ca. 86% AMX removal after 180 min. This highlights their significant potential for AMX degradation.

### 3.3. Parameters Affecting Photocatalytic Degradation of AMX

#### 3.3.1. Direct Photolysis vs. Photocatalysis

AMX solutions were exposed to sunlight without a catalyst and, after 180 min, only 29.9% of AMX was degraded (Figure 4 and Table 4). When macrocomposite photocatalysts of different compositions were added, both the reaction rate and the final percentage of degradation increased largely. The observed processes followed pseudo-first-order kinetics. In all cases, the M (0.1%)/TiO_2_ materials outperformed with respect to other with higher percentages of M. Based on this, we selected these as reference materials for the optimization of the parameters affecting the process.

**Figure 5:**
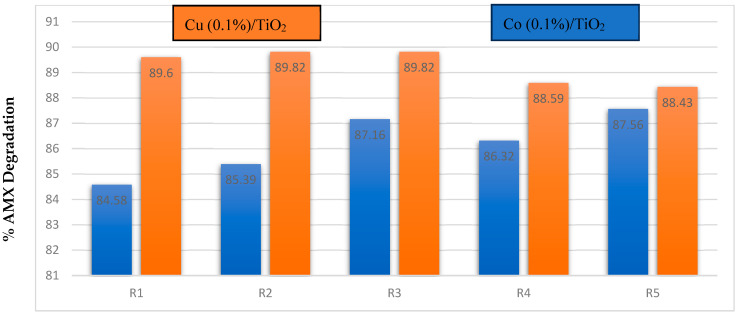
Macrocomposites reused five times with the same conditions. [AMX]₀ = 5 mg/L; photocatalyst load = 1 g/L; pH = 5.9; T = 19 °C.

Although the results are promising within the context of how to easily recycle this catalyst, it must be noted that such results cannot be easily translated into real-world applications. The long-term stability of the doped catalysts, such as the leaching of Cu and Co metals, is one of the concerns. However, stability tests of this catalyst showcase continuous degradation efficiency for amoxicillin after several cycles of photodegradation (Figure 5). This shows that the material retains integrity after time, and multiple uses directly correlate to cost effectiveness and actual use in the field.

#### 3.3.2. pH Effect

Table 5 shows the effect of the acidity (3.3 < pH < 8.6) on the degradation efficiency of AMX under sunlight by the prepared photocatalyst macrocomposites. The largest AMX percentage of photodegradation was observed at pH 3.3 for both photocatalysts.

The dependence observed on the acidity can be attributed to the relative charges of both AMX and the photocatalyst surface. The following acid-base equilibria take place at the surface of M (0.1%)/TiO_2_ [35]:

TiOH + H^+^ D TiOH_2_^+^ (1)

TiOH + HO^−^ D TiO^−^ + H_2_O (2)

Below the point of zero charge (pH_PZC_), the photocatalyst surface carries a net positive charge, while at pH > pH_PZC_, it becomes negatively charged. For the prepared macrocomposites, the pH_PZC_ values were determined to be 4.5 for Cu (0.1%)/TiO_2_ and 4.4 for Co (0.1%)/TiO_2_, indicating that the surface remains positively charged only at pH levels below approximately 4.4. Regarding AMX, three pK_a_ values have been reported [36]: 2.7 (-CO_2_H), 7.5 (-NH_2_), and 9.6 (phenolic ring). As the pH increases from 3.3 to 8.6, both the photocatalyst surface and AMX molecule acquire negative charges. The increasing electrostatic repulsion between the negatively charged surface and AMX molecules contribute to diminishing the adsorption efficiency, resulting in reduced AMX removal as pH increases. Additionally, Bougarrani et al. [37,38] observed that TiO_2_ particles tend to form agglomerates in alkaline aqueous media. These agglomerates can further contribute to the observed reduction in AMX removal rates, as they decrease the available surface area for photocatalytic activity.

#### 3.3.3. Effect of AMX Concentration

The effect of the initial AMX concentration on its photocatalytic degradation is compiled in Table 6. Degradation experiments were conducted with varying [AMX]_0_ = (1, 3, 5, 7 and 9) mg/L under sunlight irradiation for 180 min. The degradation efficiency changes from 95% for [AMX]_0_ = 1 mg/L to 75% at 3 mg/L for Cu (0.1%)/TiO_2_ and Co (0.1%)/TiO_2_. However, when the concentration of AMX was further increased to [AMX]_0_ = 9 mg/L, the degradation efficiency stabilized, reaching a plateau for both catalysts. This trend can be explained by the accumulation of organic substances on the photocatalyst surface as the initial concentration of AMX increases. Once all active sites on the photocatalyst surface are occupied, the degradation efficiency becomes constant, indicating saturation. AMX degradation with a photocatalyst loading of 1 g/L (Cu (0.1%)/TiO_2_ and Co (0.1%)/TiO_2_ achieves higher degradation at higher concentrations, particularly at pH = 5.9.

## 4. Degradation Pathways

**Figure 6:**
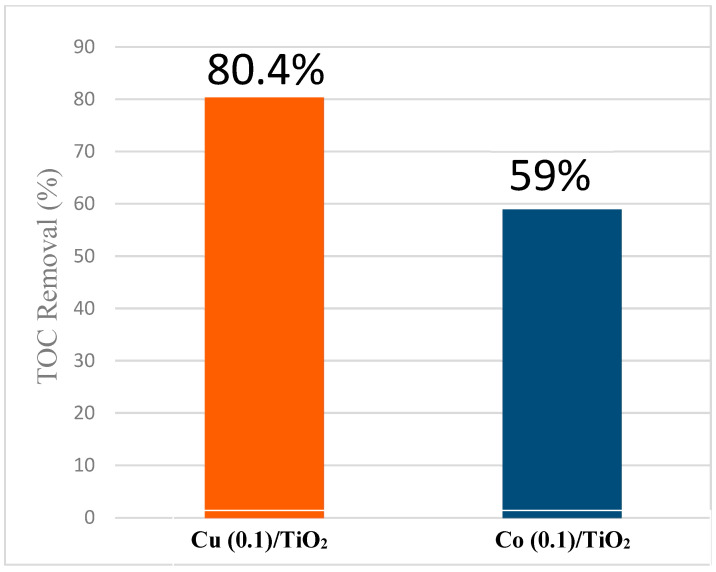
Percentage of TOC removal in the AMX degradation under sunlight irradiation using the as-synthetized M (0.1%)/TiO_2_ photocatalysts. [AMX]_0_ = 5 mg/L; catalyst dose = 1 g/L; pH = 5.9; T =19 °C.

Under sunlight irradiation, M (0.1%)/TiO_2_ macrocomposites show a high AMX removal efficiency (~85% after 180 min, Figure 4). However, AMX mineralization, measured as TOC removal, is less pronounced, achieving 80% with Cu (0.1)/TiO_2_ and 59% with Co (0.1)/TiO_2_ (Figure 6 and Figure S1, Table S1). LC-MS/MS analysis was performed to elucidate the reaction mechanism, revealing identical products for both macrocomposites; the differences lie in its reaction rate and stability.

**Figure 7:**
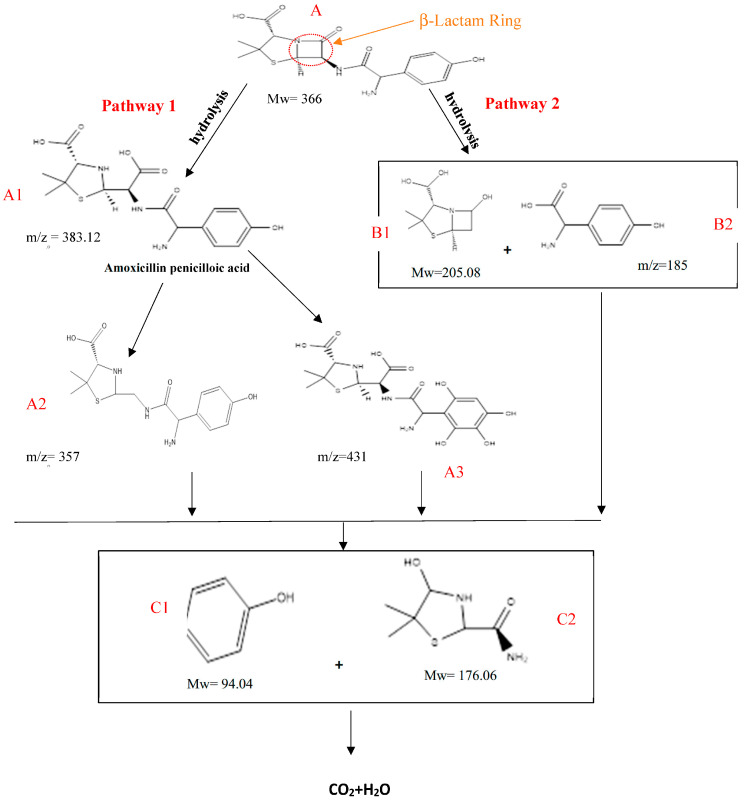
Proposed pathways for AMX degradation under sunlight irradiation using the as-synthetized macrocomposites (Cu (0.1%)/TiO_2_ and Co (0.1%)/TiO_2_.

Two primary decomposition pathways were identified (Figures S2 and S3, Figure 7). Pathway 1 involves the opening of the β-lactam ring (Figure 7), leading to the formation of amoxicillin penicilloic acid (A1, *m*/*z* = 383.12). Subsequent decarboxylation and deamination reactions generate the intermediates A2 (*m*/*z* = 357) and A3 (*m*/*z* = 431) [39].

Pathway 2 involves cleavage in the central section of AMX, producing intermediates B1 (*m*/*z* = 205.08) and B2 (*m*/*z* = 185). Intermediates C1 and C2 emerge from both pathways; specifically, C2 (*m*/*z* = 176.06) is derived from A3 and A2 through -NH-CO- bond cleavage, while phenol (C1, *m*/*z* = 95.04) originates from B2 and/or A2. As the reaction progresses, small carboxyl groups and aliphatic compounds are gradually formed, eventually mineralizing into CO_2_ and H_2_O.

The improved charge separation and transfer dynamics of Cu (0.1%)/TiO_2_ can account for its enhanced performance. Doping TiO_2_ with Cu creates extra energy levels within the bandgap of TiO_2_, working as electron traps that decrease the recombination rate of photoinduced e^−^/h^+^ pairs. This leads to an increase in reactive species (HO^•^ and O_2_^•−^) available for reaction with amoxicillin. Regarding cobalt (Co) doping, although it enhances charge separation, it may give rise to recombination centers in higher concentrations, and this can be detrimental for photocatalytic activities. Moreover, the redox potentials of Cu^+^/Cu^2+^ species are more advantageous in reactive oxygen species (ROS) production than Co^2+^/Co³^+^, leading to the higher mineralization efficiency that we observed [40,41,42].

## 5. Conclusions

This study focused on preparing and characterizing efficient macrocomposites (in the form of pellets) for the abatement of water pollutants, specifically targeting the photodegradation of AMX under sunlight irradiation. AMX was selected as a model compound due to its widespread use and frequent detection in aquatic environments. Recent reviews have highlighted the environmental risks associated with AMX contamination and the effectiveness of photocatalytic degradation as a remediation strategy [43,44].

Kaolin clay and TiO_2_-P25 composites were doped with Cu and Co using the incipient wetness impregnation method. The resulting macrocomposites were characterized using SEM-EDS, XRD, XRF, textural property analysis, and their lixiviation through ICP-MS.

The effects of medium acidity, AMX concentration, catalyst percentage, and reuse on AMX degradation under sunlight were monitored via UV–Vis spectrophotometry and UV–Vis HPLC analysis at 226 and 272 nm. A pseudo-first-order kinetic model fitted the observed data for AMX photodegradation. Optimal formulations, Cu (0.1%)/TiO_2_ and Co (0.1%)/TiO_2_, achieved up to 95% photodegradation of 5 mg·L^−1^ AMX in 180 min at pH 5.9 with a catalyst load of 1 g·L^−1^. While both doped macrocomposites exhibited similar photodegradation percentages, the Cu-doped composite demonstrated a slightly faster reaction rate and higher TOC removal (80.4%) compared to the Co-doped composite (59%) after 180 min of sunlight irradiation under the same conditions (*vide supra*). The results indicate that Cu and Co-doped kaolin clay and TiO_2_-P25 composites are effective photocatalysts for AMX degradation in water under solar irradiation.

The main photodegradation intermediates were identified via LC-MS, revealing consistent intermediates regardless of the doped metal, and a reaction mechanism was proposed. These cost-effective and durable macrocomposites exhibit good durability with efficient and consistent recycling over more than five cycles, making them ideal candidates for mitigating antibiotic pollution in water-treatment facilities.

## Figures and Tables

**Table 1:** Chemical analysis Cu (0.1%)/TiO_2_, Co (0.1%)/TiO_2_ pellets measured by XRF.

%	TiO_2_	SiO_2_	Al_2_O_3_	Fe_2_O_3_	K_2_O	SO_3_	CO_2_	CaO	CuO	CoO	NiO	Cr_2_O_3_	V_2_O_5_
Cu	92.7	2.7	2.1	0.23	0.18	0.11	1.7	0.039	0.14	---	0.013	0.067	---
Co	93.5	2.9	2.2	0.22	0.19	0.12	0.51	0.022	---	0.070	---	---	0.25

**Table 2:** BET parameters and textural properties of Cu (0.1%)/TiO_2_ and Co (0.1%)/TiO_2_ photocatalysts measured by N_2_ adsorption–desorption. TiO_2_-P25, BET surface area = 51 m^2^·g^−1^.

Photocatalyst	Cu (0.1%)/TiO_2_		Co (0.1%)/TiO_2_	
Property	Ref. [32]	This Work	This Work	Ref. [33]
SBET (m^2^·g^−1^)	51 ^a^	47.0 ± 0.1	45.5 ± 0.1	22.8 ^a^
Constant C		139.5	119.7	
Pore volume (cm^3^·g^−1^)		0.101	0.095	0.033
Average pore width (4 V/A) (nm)		96 ^b^	94 ^b^	65 ^c^
Average particle size (A)		127.5	131.7	
Median pore width (nm)		0.7758	0.7788	

^a^ Hydrothermal synthesis; ^b^ D-H desorption; ^c^ average pore diameter.

**Table 3:** Dark adsorption of the AMX of the as-synthetized photocatalysts. [AMX]_0_ = 5 mg/L; photocatalyst load = 1 g/L; pH = 5.9; T = 19 °C.

Time/min	15	30	60	120	180
% AMX adsorption on Cu (0.1)/TiO_2_	0.54	2.56	5.42	8.40	13.82
% AMX adsorption on Co (0.1)/TiO_2_	3.05	5.51	2.50	14.51	18.01

**Table 4:** Observed apparent first-order rate constant and AMX photocatalytic degradation under sunlight using the prepared M (X%)/TiO_2_ macrocomposites (pellets). [AMX]_0_ = 5 mg/L; photocatalyst load = 1 g/L; pH = 5.9; T = 19 °C.

Photocatalyst	k·10^3^/min^−1^	% Removal at 120 min	% Removal at 180 min
Photolysis	39.6	24.2	31.1
Cu (0.1%)/TiO_2_	152.5	86.7	86.4
Cu (0.5%)/TiO_2_	102.5	65.3	79.2
Cu (1.0%)/TiO_2_	128.4	88.0	87.6
Co (0.1%)/TiO_2_	107.2	83.4	84.4
Co (0.5%)/TiO_2_	76.9	81.7	82.3
Co (1.0%)/TiO_2_	28.6	56.7	69.1

**Table 5:** Influence of pH on the photocatalytic degradation of AMX under sunlight. [AMX]_0_ = 5 mg/L; catalyst dose = 1 g/L; T =19 °C.

Photocatalyst	Property	pH		
		3.3	5.9	8.6
Cu (0.1%)/TiO_2_	k·10^3^/min^−1^	64.1	152.5	9.2
	% removal at 180 min	90.9	86.4	86.1
Co (0.1%)/TiO_2_	k·10^3^/min^−1^	133.7	107.2	37.0
	% removal at 180 min	89.5	84.4	76.8

**Table 6:** The effect of [AMX]_0_ on its photocatalytic degradation. AMX percentage removal, after 180 min under sunlight, is reported. Catalyst load 1 g/L; pH = 5.9; T =19 °C.

[AMX]_0_/mg·L^−1^	1	3	5	7	9
Cu (0.1%)/TiO_2_	95.4	73.9	86.4	87.8	82.0
Co (0.1%)/TiO_2_	96.4	75.3	84.4	66.9	82.0

## Data Availability

The original contributions presented in this study are included in the article/Supplementary Material. Further inquiries can be directed to the corresponding author.

## Supplementary Materials

The following supporting information can be downloaded at: https://www.mdpi.com/article/10.3390/ma18071394/s1, Figure S1: Degradation %, COD and BOD of Amoxicillin Over Time using Cu (0.1)/TiO_2_ and Co (0.1)/TiO_2_. [AMX]_0_ = 15 mg/L; catalyst dose = 1 g/L; pH = 5.9; T = 19 °C; Figure S2: Chromatogram of Amoxicillin; Figure S3: Mass spectra of the byproducts during the photocatalytic degradation of Amoxicillin using Cu (0.1%)/TiO_2_ under sunlight. Table S1: Residual COD, BOD and BOD/COD Ratio for Cu (0.1)/TiO_2_, Co (0.1%)/TiO_2_ Over Time. [AMX]_0_ = 15 mg/L; catalyst dose = 1 g/L; pH=5.9; T = 19 °C.
