# Supplementary material for: Treatment of Aqueous Amoxicillin Solutions with Sunlight Using a Pelletized Macrocomposite Photocatalyst
Source: Materials (Basel). 2025 Mar 21;18(7):1394. doi: 10.3390/ma18071394 (PMC11990014; doi:10.3390/ma18071394)
Supplement: Supplementary file 1 [file materials-18-01394-s001.zip › materials-3501321-supplementary.pdf]

Supplementary material

Treatment of Aqueous Amoxicillin Solutions with Sunlight Using a Pelletized Macrocomposite Photocatalyst

Saad Slimani Tlemcani, Zenydia Marín, J. Arturo Santaballa and Moisés Canle *

React! Group, Department of Chemistry, Faculty of Sciences & CICA, Universidade da Coruña, E-15071 A Coruña, Spain; saad.slimanitlemcani@udc.es (S.S.T.); zenydia.marin@udc.es (Z.M.); arturo.santaballa@udc.es (J.A.S.)
* Correspondence: moises.canle@udc.es

Table S1: Residual COD, BOD and BOD/COD Ratio for Cu(0.1)/TiO_2 , Co(0.1%)/TiO2 Over Time. $[AMX]_0$ = 15 mg/L; catalyst dose = 1 g/L; pH=5.9; T =19 °C.

Time (min)	Cu(0.1%)/TiO_2 - COD (mg/L)	Cu(0.1%)/TiO_2 - BOD (mg/L)	BOD/COD Ratio	Co(0.1%)/TiO_2 - COD (mg/L)	Co(0.1%)/TiO_2 - BOD (mg/L)	BOD/COD Ratio
0	51.3	19.6	0.38	51.3	19.6	0.38
60	35.9	13.7	0.38	40.0	15.7	0.39
120	20.5	7.8	0.38	30.0	11.8	0.39
180	10.3	3.9	0.37	21.0	8.0	0.38

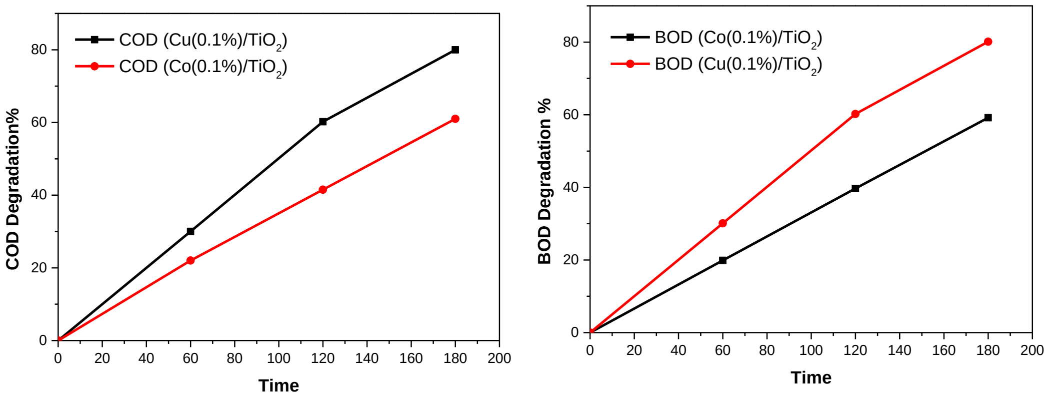

*

Figure S1: Degradation %, COD and BOD of Amoxicillin Over Time using Cu(0.1)/TiO_2 and Co(0.1)/TiO_2.$[AMX]_0$ = 15 mg/L; catalyst dose = 1 g/L; pH=5.9; T =19 °C.

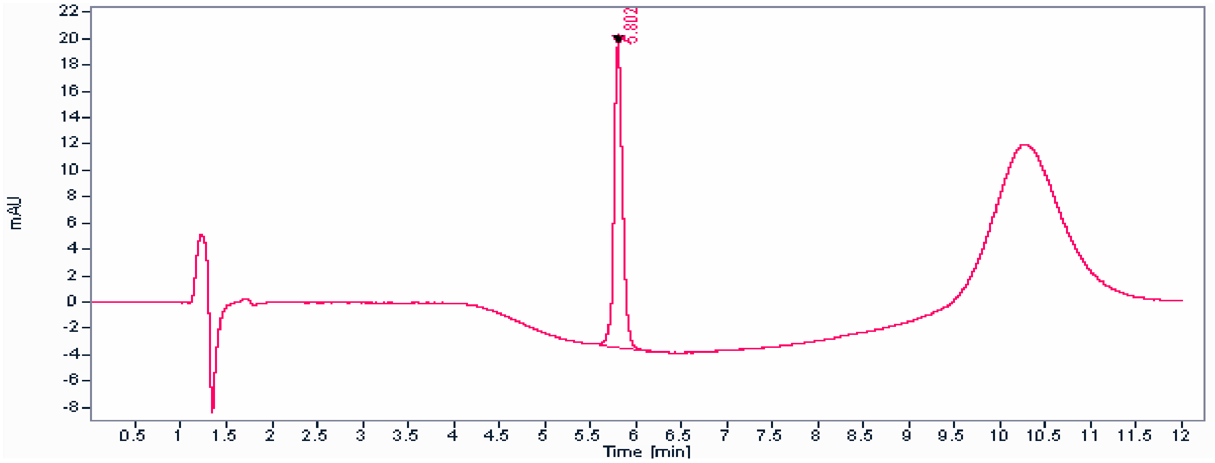

Figure S2: Chromatogram of Amoxicillin

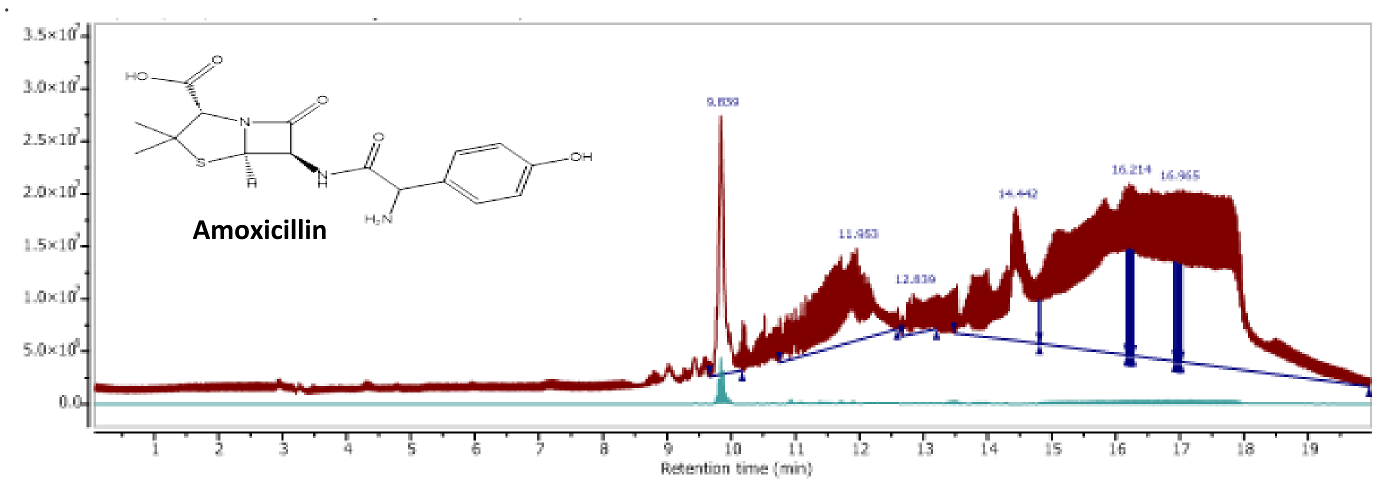

Molecule	Molecule Info	Match	Match Score	Similarity	MS Purity	RT	Scan	Purity	MS/MS Match Score	Adduct/Loss	Error (ppm)	Error (mDa)	Predicted m/z	Matched m/z
1 $C_{16}H_{19}N_3O_5S$	Name: —	Yes	0.998	0.998	0.151	9.84	861	100.00%	—	H+ / —	1.383	0.506	366.1118	366.1123

Label: —

Formula: $C_{16}H_{19}N_3O_5S$

Monoisotopic Mass: 365.1045

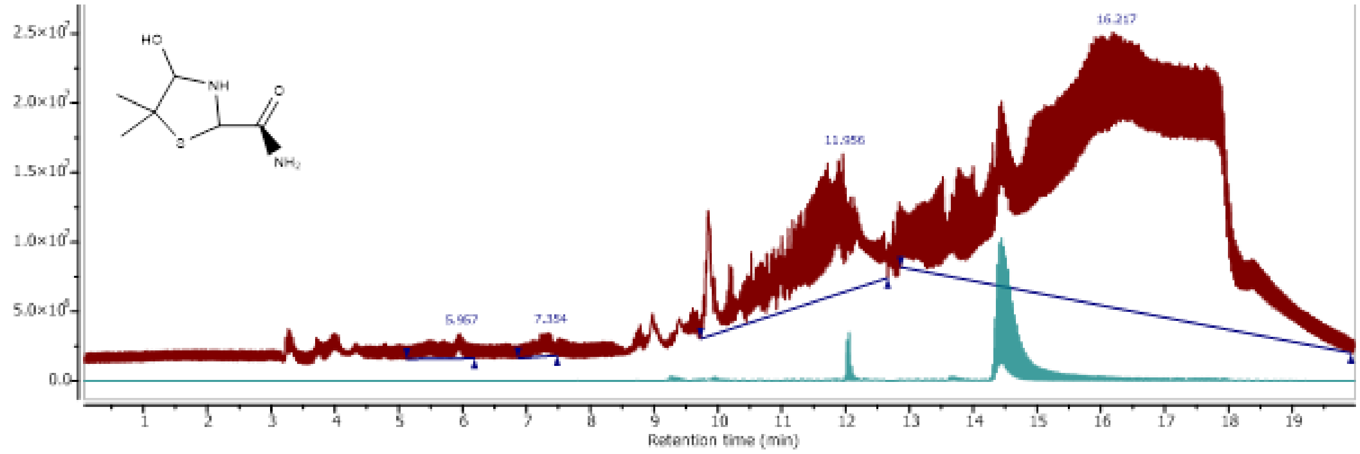

	Molecule	Molecule Info	Match	Match Score	Similarity	MS Purity	RT	Scan	Purity	MS/MS Match Score	Adduct /Loss	Error (ppm)	Error (mDa)	Predicted m/z	Matched m/z
1	C6H12N2O2S	Name: — Label: — Formula: C6H12N2O2S Monoisotopic Mass: 176.0619	Yes	0.974	0.974	0.604	14.44	1266	90.21 %	—	2Na+ / H+	1013.115	233.033	230.0160	230.2490

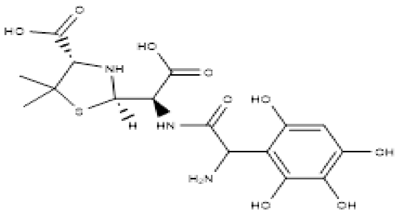

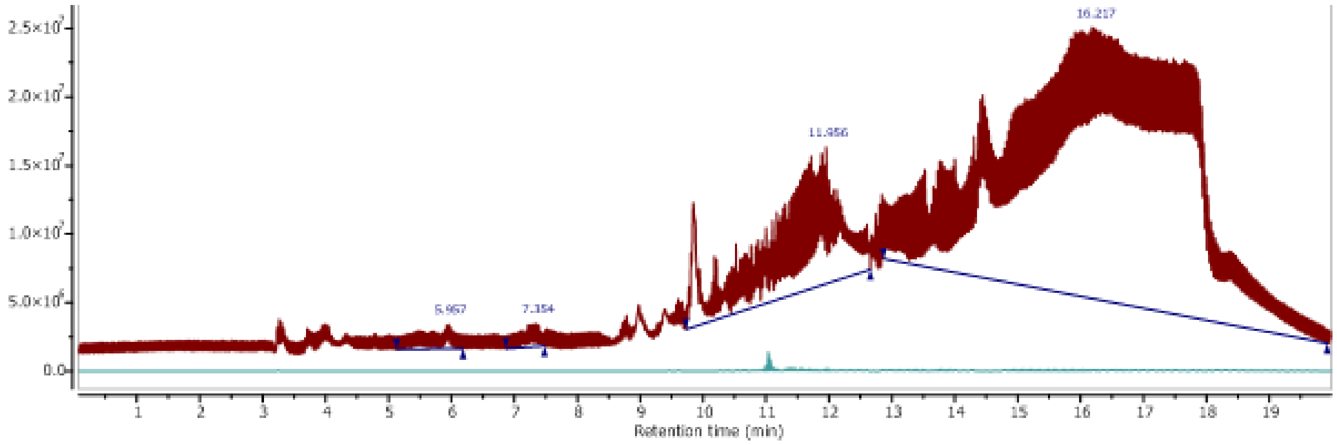

	Molecule	Molecule Info	Match	Match Score	Similarity	MS Purity	RT	Scan	Purity	MS/MS Match Score	Adduct/Loss	Error (ppm)	Error (mDa)	Predicted m/z	Matched m/z
1	C16H21N3O9S	Name: —<br>Label: —<br>Formula: C16H21N3O9S<br>Monoisotopic Mass: 431.0999	Yes	0.983	0.983	0.220	11.04	966	25.61%	—	H+ / —	402.063	173.734	432.1071	432.2809

FigureS3: Mass spectra of the byproducts during the photocatalytic degradation of Amoxicillin using Cu(0.1%)/TiO2 under sunlight.
